# Clinical implications of gait analysis in the rehabilitation of adult patients with "Prader-Willi" Syndrome: a cross-sectional comparative study ("Prader-Willi" Syndrome vs matched obese patients and healthy subjects)

**DOI:** 10.1186/1743-0003-4-14

**Published:** 2007-05-10

**Authors:** Luca Vismara, Marianna Romei, Manuela Galli, Angelo Montesano, Gabriele Baccalaro, Marcello Crivellini, Graziano Grugni

**Affiliations:** 1 Physical Medicine and Rehabilitation Unit and Clinical Lab for Gait Analysis and Posture, Istituto Scientifico Ospedale San Giuseppe, Verbania, Italy; 2 Bioengineering Department, Politecnico di Milano, Italy; 3 Unit of Auxology, Istituto Scientifico Ospedale San Giuseppe, Verbania, Italy; 4 SOMA – School of Osteopathic Manipulation, Milano, Italy

## Abstract

**Background:**

Being severely overweight is a distinctive clinical feature of Prader-Willi Syndrome (PWS). PWS is a complex multisystem disorder, representing the most common form of genetic obesity. The aim of this study was the analysis of the gait pattern of adult subjects with PWS by using three-Dimensional Gait Analysis. The results were compared with those obtained in a group of obese patients and in a group of healthy subjects.

**Methods:**

Cross-sectional, comparative study: 19 patients with PWS (11 males and 8 females, age: 18–40 years, BMI: 29.3–50.3 kg/m^2^); 14 obese matched patients (5 males and 9 females, age: 18–40 years, BMI: 34.3–45.2 kg/m^2^); 20 healthy subjects (10 males and 10 females, age: 21–41 years, BMI: 19.3–25.4 kg/m^2^). Kinematic and kinetic parameters during walking were assessed by an optoelectronic system and two force platforms.

**Results:**

PWS adult patients walked slower, had a shorter stride length, a lower cadence and a longer stance phase compared with both matched obese, and healthy subjects. Obese matched patients showed spatio-temporal parameters significantly different from healthy subjects.

Furthermore, Range Of Motion (ROM) at knee and ankle, and plantaflexor activity of PWS patients were significantly different between obese and healthy subjects. Obese subjects revealed kinematic and kinetic data similar to healthy subjects.

**Conclusion:**

PWS subjects had a gait pattern significantly different from obese patients. Despite that, both groups had a similar BMI. We suggest that PWS gait abnormalities may be related to abnormalities in the development of motor skills in childhood, due to precocious obesity. A tailored rehabilitation program in early childhood of PWS patients could prevent gait pattern changes.

## Background

Obesity is a pathological condition associated with impairment in skeletal statics and dynamics. Excess weight is able to induce negative effects on several common daily movements, such as standing up, bending, walking and running [1,2]. The analysis of gait pattern of obese children shows a more flat-footed weight acceptance period in stance phase and greater out-toeing of the foot in the gait cycle [3]; moreover, obese children walk with a significanlty lower peak knee flexion angle during early stance but they did not show any change in sagittal plane knee moment [4]. As far as obese adult patients are concerned, obese males display a gait pattern similar to healthy subjects but some of the temporal and angular components seem different from those observed in non obese individuals, mainly because of the excessive adipose tissue inside their thighs [5]. Furthermore, it has been suggested that humans reorganize their neuromuscular function when walking with excessive weight, in order to increase ankle muscle function, plantarflexion torque and ankle power [6].

Severe overweight is a distinctive clinical feature of Prader-Willi Syndrome (PWS). PWS is a complex multisystem disorder, representing the most common form of genetic obesity. The genetic basis is a paternal derived deletion within 15q11–q13 (70–75% of cases), a maternal uniparental disomy of chromosome 15 (UPD15) (20–25%), or a defect in the imprinting center (2%) [7]. Children with PWS usually become obese during early childhood [8], as a consequence of an insatiable appetite for food and excessive food intake. Obesity associated with PWS is often massive and may subjects exceeded their ideal body weight by more than 200% [9]. Other typical PWS characteristics that may interfere with gait pattern include muscular hypotonia, short stature, small hands and/or feet (acromicria) and scoliosis. Hypotonia is nearly uniformly present and gradually improves with age. Nevertheless, adults remain mildly hypotonic with decreased fat free mass [10].

Growth failure is a recognized feature of the PWS patients [11]. Short stature appears to be caused by the lack of the pubertal growth spurt and the presence of a GH/IGF-I axis deficiency [12], probably due to hypothalamic dysfunction [13]. Final height of PWS subjects ranged from 142–150 cm for females and 152–162 cm for males [10]. Dysmorphic features include small narrow hands and/or short feet, with an average adult foot length of 20.3 cm for females and 22.3 cm for males [14]. Scoliosis generally becomes more evident during adolescence and can contribute to the short stature. In addition to scoliosis, other major orthopedic findings for PWS patients are: flat feet (47%), knock knees (19%), hip dysplasia (10%), osteoporosis (9%) and patellofemoral instability (7%) [15].

No previous studies have analyzed the PWS subjects' movement ability in daily activity such as walking.

Taken into consideration the peculiar clinical picture of patients with PWS, aim of our study was to characterize the gait pattern of these subjects by using 3D-Gait Analysis. The results were compared with those obtained in a group of healthy obese subjects and in a group of healthy subjects.

## Methods

### Patients

Nineteen patients with PWS, 11 males and 8 females, aged 18–40 years, were admitted to the study (Table 1). These subjects were periodically hospitalized at "Istituto Scientifico Ospedale S. Giuseppe" and they underwent clinical assessments and attended a rehabilitation program. All patients showed the typical PWS clinical phenotype [16]. Cytogenetic analysis was performed in all subjects; 13 out of them had interstitial deletion of the proximal long arm of chromosome 15 (del15q11–q13). Moreover, uniparental maternal disomy for chromosome 15 (UPD15) was found in 6 individuals. Seventeen subjects were obese and 2 overweight. Mean Body Mass Index (BMI) and Standard Deviation (± SD) were 41.3 ± 6.0 kg/m^2 ^(range 29.3–50.1 kg/m^2^). Standing height was determined by a Harpenden Stadiometer and expressed as centimeters. Body weight was measured to the nearest 0.1 kg on a precision digitale scale, while the subject was wearing only shorts and T-shirt. All patients showed short stature for genetic background (Table 1).

**Table 1:** Clinical and laboratory data of patients with Prader-Willi syndrome

Patients	sex	Age (yr)	Karyotype*	Height (cm)	Weight (kg)	BMI (kg/m^2^)
1	M	25.0	UPD15	155.5	71.0	29.3
2	M	40.0	UPD15	149.8	94.4	42.0
3	M	30.3	de1l5	157.3	103.0	41.6
4	M	30.1	del15	150.8	94.1	41.3
5	M	18.4	UPD15	154.9	79.5	33.1
6	M	23.6	del15	165.5	115.6	42.2
7	M	17.7	del15	157.4	118.4	47.8
8	M	22.6	del15	159.0	117.8	46.5
9	M	20.2	del15	160.0	128.9	50.3
10	M	18.0	del15	163.0	124.5	46.8
11	M	29.9	del15	161.0	108.7	41.9
12	F	23.4	UPD15	147.0	78.6	36.3
13	F	29.0	UPD15	142.8	86.5	42.4
14	F	22.7	del15	148.8	86.1	38.8
15	F	23.6	del15	142.5	88.2	43.4
16	F	33.1	del15	149.0	65.5	29.5
17	F	28.1	UPD15	153.7	118.5	50.1
18	F	33.1	del15	144.0	90.1	43.4
19	F	19.2	del15	147.5	84	38.6
*Mean *± *SD*		*25.7 *± *6.1*		*153.1 *± *6.9*	*97.5 *± *19*	*41.3 *± *6.0*

*del15: interstitial deletion of the proximal long arm of chromosome 15; UPD15: uniparental maternal disomy for chromosome 15.

Two different groups of subjects were specifically recruited for this study and served as controls (Table 2). The first group included 14 obese patients (mean BMI = 39.2 ± 3.25 kg/m^2^, range from 34.3 to 45.2), 5 males and 9 females, aged 18–40 years. The second group included 20 healthy subjects, 10 males and 10 females, aged 21–41 years, with a BMI ranging from 19.3 to 25.4 (mean BMI for healthy subjects was 21.4 ± 2.2 kg/m^2^). All PWS and control obese patients were found with normal values in main laboratory tests, including adrenal and thyroid function.

**Table 2:** Clinical characteristics of the study groups

Groups	Sample size	Age (years)	Height (cm)	Weight (kg)	BMI (kg/m^2^)
PWS	19	25.7 ± 6.1	153.1 ± 6.9	97.5 ± 19	41.3 ± 6
Obese	14	29.4 ± 7.9	160.4 ± 7.1	101.2 ± 12.9	39.2 ± 3.25
Healthy	20	30.2 ± 5.2	170.6 ± 5.6*	62.6 ± 9.3*	21.4 ± 2.2*

Data are expressed as mean ± SD. *p < 0.0001 *versus *PWS and obese patients.

The study protocol was approved by the Ethical Committee of the "Istituto Auxologico Italiano". Written informed consent was obtained by the parents and, when applicable, the patients.

### Protocol

All the subjects performed a three-dimensional Gait Analysis (GA) assessment at the Movement Analysis Lab of "Istituto Scientifico Ospedale S. Giuseppe". GA was comprised in the clinical assessment that all the ambulant patients have during the hospitalization. The Lab was equipped with an optoelectronic system with 6 cameras (460 Vicon, UK) working at 100 Hz and two force platforms (Kistler, CH). Twenty-three passive markers were placed on the subject's body according to the Davis' protocol [17].

Each subject was instructed to walk on a walkway ten meters long at their preferred speed. In order to reach the first platform with the right foot and the second platform with the left foot, for each subject the starting point was identified and located on the walkway. For obese and healthy subjects the acquisition of dynamic data for both legs in a single trial was possible; for some PWS subjects it was not possible, because of the short step length due to their short lower limbs. In these cases, dynamic data of left and right leg were separately assessed.

Then, for each patient at least five trials with kinematic and kinetic data were collected and comparing the different plots of kinematic and kinetics were extracted three trials able to evidence the same gait pattern (from kinematics and kinetics point of view) with the same gait speed. These trials were considered for the following analysis. The data were considered repeatable according to the values of gait velocity. Cadence (steps min^-1^), duration of stance phase (as % of gait cycle), duration of single support (as % of gait cycle), stride length (m) and walking speed (m s^-1^) were considered as spatio-temporal parameters. In order to take into account the variability in height between the three groups (Table 2), stride length and walking speed were normalized to the subject's height; normalized values were considered for statistical analysis.

For PWS patients' gait pattern characterization, kinematic and kinetic parameters were identified and then extracted from each subject's trial. For hip and knee joint, Range Of Motion (ROM) on sagittal plane was considered as the most important parameters for the analysis of articular mobility. ROM was calculated as difference between absolute maximum (MAX) and absolute minimum (MIN) of the curve of joint movement. Beside this, the mean values of MAX and MIN were considered. For ankle joint, in addition to ROM on sagittal plane, peak of plantarflexion, peak of dorsiflexion in swing phase and foot progression mean values during the gait cycle were analysed. Foot progression represents the rotation of the foot (external/internal rotation) in respect to the walking direction and is defined as the angle formed with the line of progression and the segment connecting the marker on the V metatarsal joint and the marker on external malleulus. Peak of ankle dorsiflexion moment and peak of ankle power normalized both to the subject's weight and to the walking velocity were calculated as kinetic parameters in order to investigate the push-off ability during the propulsive phase of the gait cycle (terminal stance).

The results are expressed as mean ± SD. Statistical analysis was performed by t-test for unpaired data with Bonferroni correction, and using analysis of variance for parametric or nonparametric (Kruskall-Wallis and Mann-Whitney) data, where appropriate; *P *values less than 0.05 were considered significant.

## Results

Most of the spatio-temporal parameters were significantly different between the three groups (Table 3). Compared with obese individuals, PWS patients data differed more markedly from those calculated for healthy subjects.

**Table 3:** Spatio-temporal parameters of the study groups

Groups	PWS	Obese	Healthy
Cadence (steps min^-1^)	111.76 ± 9.12†	115.57 ± 4.60	117.84 ± 4.80
Stance (% gait cycle)	63.88 ± 2.47*	62.22 ± 1.28‡	60.07 ± 1.40
Single Support (% gait cycle)	35.81 ± 3.94*	37.76 ± 1.34‡	39.91 ± 1.48
Normalized Stride Length	0.67 ± 0.07*	0.76 ± 0.05‡	0.80 ± 0.04
Normalized Walking Speed (s^-1^)	0.63 ± 0.09*	0.73 ± 0.06‡	0.78 ± 0.06

Data are expressed as mean ± SD. Stride length and walking speed were normalized to the subject's height.

*p < 0.0001 *versus *obese patients and healthy subjects, †p < 0.002 *versus *obese patients and healthy subjects; ‡*p *< 0.02 *versus *healthy subjects.

PWS subjects walked with a 5% reduced cadence, with a 6.3% longer stance phase duration, a 10% reduced single support phase, with a 16.25% shorter normalized stride length and at a 19% slower normalized velocity, compared to healthy controls. Moreover, PWS patients had a 3% reduced cadence, their stance phase lasted 2% more, their single support was 5% reduced, the normalized stride length was 11.8% shorter and normalized walking speed was 14% reduced, compared to obese subjects. Furthermore, cadence of obese partecipants was 1.9% lower than that of normal, stance duration lasted 3.6% more than normal, the reduction of normalized stride length was 5% and they walked with a 6.4% reduced normalized velocity, compared to healthy subjects.

Joint kinematic parameters revealed significant differences between PWS patients and both healthy and obese subjects in ROM at knee and ankle parameters (Table 4), with the exception of ROM at hip. In particular, PWS patients showed statistically significant reduced sagittal plane ROM at knee and ankle in comparison both with obese and healthy subjects. In addition, kinematic parameters of obese patients were similar to those found in healthy individuals, apart from foot progression.

**Table 4:** Kinematic parameters of the study groups

Groups	PWS	Obese	Healthy
ROM at Hip (°)	46.19 ± 5.4	44.45 ± 4.1	45.92 ± 3.25
ROM at Knee (°)	56.11 ± 8.24 ‡	60.12 ± 6.10	61.23 ± 4.02
ROM at Ankle (°)	25.06 ± 3.65 *	29.81 ± 6.88	31.90 ± 4.81
Peak of ankle plantarflexion (°)	-8.31 ± 5.87 *	-15.85 ± 6.61	-18.98 ± 6.19
Peak of ankle dorsiflexion in swing (°)	15.63 ± 6.59 *	5.08 ± 2.36	4.19 ± 3.53
Foot progression (°)	-16.64 ± 8.92 *	-13.73 ± 5.19 †	-6.88 ± 3.96

Data are expressed as mean ± SD (in degrees, °). *p < 0.0001 *versus *obese and healthy partecipants; ‡ p < 0.001 *versus *obese and healthy participants; †*p *< 0.001 *versus *healthy subjects.

The difference in ROM at knee between PWS and healthy subjects was due more to a reduced peak of flexion (MAX_PWS _= 53.84° ± 7.34°, MAX_healthy _= 61.35° ± 4°; p < 0.0001) than to a limited knee extension (MIN_PWS _= -2.27° ± 5.94°, MIN_healthy _= 0.12° ± 3.06°; p = 0.035). The same differences were found between PWS and obese subjects (MAX_obese _= 58.23° ± 4.4°: p = 0.008; MIN_obese _= -1.88° ± 4.15°: p > 0.05). PWS and obese individuals revealed an hyperextended knee in stance phase that was not present in knee pattern of healthy subjects. Moreover, knee pattern of PWS subjects didn't demonstrated to be notably flexed during the gait cycle.

Compared to healthy subjects and obese patients' gait pattern, ankle's parameters showed a reduced ROM and a more dorsiflexed position for PWS subjects both in stance and in swing phase of the gait cycle. A lower peak of plantarflexion (MIN_PWS _= -8.31° ± 5.87° *versus *MIN_obese _= -15.85° ± 6.61° (p < 0.0001) and *versus *MIN_healthy _= -18.98° ± 6.19° (p < 0.0001)) determined a reduced ROM at the ankle rather than the peak of dorsiflexion (MAX_PWS _= 16.75° ± 5.89° *versus *MAX_obese _= 13.95° ± 3.34°, p = 0.003) and *versus *MAX_healthy _= 12.91° ± 2.97°, p < 0.0001)). Moreover, the PWS subjects' foot was more externally rotated during the entire gait cycle in respect to both healthy and obese subjects.

Gait pattern of obese subjects revealed to be similar to that found for healthy subjects. The only statistically significant difference was related to the position of the foot in respect to the ground: obese subjects walked with a more externally rotated foot compared with healthy subjects (mean foot progression_obese _= -13.73° ± 5.19°, mean foot progression_healthy _= -6.88° ± 3.96°, p < 0.001). ROM at Hip, Knee and Ankle on sagittal plane didn' show statistically significative difference between obese and healthy partecipants (obese *versus *healthy subjects; ROM hip: p = 0.17, ROM knee: p = 0.39; ROM ankle: p = 0.113).

With regard to kinetic parameters, PWS values were lower than those obtained in obese and healthy subjects (Table 5), particularly for ankle joint power. Furthermore, obese patients showed slightly higher values in respect to healthy subjects, but the differences were not statistically significant.

**Table 5:** Kinetic parameters of the study groups

Groups	PWS	Obese	Healthy
Peak of plantarflexion moment (N s kg^-1^)	1.07 ± 0.22*	1.20 ± 0.14	1.13 ± 0.13
Peak of ankle generated power (W s kg^-1 ^m^-1^)	1.95 ± 0.53†	2.69 ± 0.5	2.57 ± 0.4

Data are expressed as mean ± SD. Peak of plantarflexion moment and Peak of ankle power were normalized to subject's weight and velocity. *p < 0.01 *versus *obese and healthy subjects; †*p *< 0.001 *versus *obese and healthy subjects.

## Discussion

Morbility and mortality of PWS are mainly related to severe obesity. Hypothalamic dysfunction is a recognized cause of compulsive appetite leading PWS patients to develop obesity [18]. Moreover, physical activity of PWS is generally reduced, as a consequence of deficits in muscle mass, physical strength, and agility [19]. Physical inactivity may significantly contribute to the development and the maintenance of obesity. Similarly to essential obesity, altered skeletal statics and dynamics caused by fat mass accumulation may in turn worsen physical performances of patients with PWS. On the other hand, PWS shows peculiar dysmorphic features that may interfere with physical activity, such as muscular hypotonia, short stature, acromicria, and scoliosis. Therefore, in this study we have investigated whether gait pattern of adult subjects with PWS was different from those observed in patients with obese patients and in healthy subjects.

The analysis of spatio-temporal parameters shows that PWS subjects are slower, have shorter stride length as well as more prolonged stance phase and reduced single support phase compared with both obese and healthy subjects. This motor strategy is likely to be aimed at avoiding overloading on one single limb and maintaining the weight on both the limbs. The presence of small feet in PWS subjects may be an additional factor explaining the decrease in the single support phase compared to obese controls. Furthermore, dorsal kyphosis in PWS subjects [20] that anteriorly tilt the pelvis associated with excessive fat on the abdomen can be responsible for forward displacement of the center of gravity creating instability during standing and walking.

The self-selected walking speed of obese subjects is 1.17 ± 0.10 m/sec; Browning et al [21] reported that the velocity that minimizes the energy cost per distance for a group of obese women was 1.2 m/s, similar to what was found in this study and elsewhere [22,23]. This means that, when asked to walk at their preferred speed, obese patients walk at a velocity that minimizes the energy cost. Other studies carried out on obese patients [5,6] reported 1.09 ± 0.14 m/sec and 1.29 ± 0.15 m/sec as free-selected speed. The difference found in these studies are likely related to the variability in the obese population or different methodology in data collection, such as walking outdoor or on a treadmill. Furthermore, the patients analysed in the mentioned studies were older than ours (38.92 ± 6.42 and 39.5 ± 8.8 versus 29.4 ± 7.9 years) and in the study of Spyropoulos et al [5] BMI values were not reported.

Cadence does not show any difference between obese and healthy subjects, whereas a prolonged (p < 0.001) stance duration and a reduced (p < 0.001) single support duration revealed a gait pattern more involved in balance control for obese patients.

Kinematic and kinetic parameters display a gait pattern that is peculiar for PWS patients. The only common aspect with obese controls is the presence of the external rotation of the foot during the entire gait cycle (PWS = -16.6° ± 8.9°, obese = -13.7° ± 5.2°, p = 0.169). An externally rotated foot could be due both to the presence of excessive adipose tissue inside the thighs, as previously suggested [5] and to the presence of flat foot due to the overload. Recent studies of the load distribution on the sole of the foot [24] in young obese patients during standing and walking, revealed a relevant increase in the foot surface in contact with the ground. This would predispose to the development of a pathological foot, as demonstrated by the greater incidence of flat foot in obese children [25]. Particularly, in PWS patients, abnormalities in foot loading and hypotonia may be responsible for changes in the foot structure and can cause the collapse of the longitudinal arc and a decrease in foot functionality.

Except for hip joint, motion of the knee and ankle joints are significantly different in PWS subjects compared to both obese and healthy subjects (Table 3). Range of motion of both knee and ankle of PWS are significantly reduced compared with obese and healthy subjects. More specifically, the ankle seems to show the most different pattern in respect to obese patients and healthy subjects, and is likely to be the landmark of the pathological gait strategy of PWS patients.

In relation to knee joint, the 63.16% (12/19) of PWS patients presents an hyperextended knee during stance phase, that is likely due to the excessive load that the knee must support during the stance phase. In normal gait the load of the body is supported by the muscle activity of the leg, but in an overweight situation a more pronounced knee extension can reduce the activity of quadriceps and hamstrings. Furthermore, muscular hypotonicity observed in PWS patients is likely to be the only stategy that allows them to bear their weight while extending the knee. This finding is found in a lower percentage of obese subjects (35.7% – 5/14): the muscles of these patients are able to support the load without extending the knee.

Obese subjects kinematic and kinetic data show a gait pattern similar to that of healthy subjects; the only difference is in spatio-temporal parameters and the more externally rotated foot for obese patients. These results support that obesity does not determine major and immediate changes in the learned motor strategy in young adult obese patients. Many obese patients older than those recruited for this study often show articular problems and pathological gait pattern [26,27] that could be due to the progressive effect of excessive joint loads over the years. Then, the effect of obesity on joint biomechanics is not immediate, but progressive.

The kinetic data of PWS subjects' ankle show a reduced plantarflexor activity and based on these data, the presence of hypotonia in PWS subjects [10] may explain the clinically relevant decrease in push-off ability.

Based on kinematic and kinetic results, PWS gait pattern strongly differs from obese subjects, despite both groups have similar BMI (Table 2).

## Conclusion

By using instrumented GA the gait pattern of PWS subjects was quantitatively characterized and it resulted different from those of obese and healthy subjects, mainly as concern knee and ankle joints. An hypothesis explaining PWS gait abnormalities may be the changes in the development of motor skills in early childhood. It was mentioned before that during the first year of life PWS newborns are hypotonic and they develop their obesity when they are 2–3 years old. It is well known that these two conditions affect the development of motor and functional skills that children usually learn at that age [28]: PWS children's ability in sitting, kneeling, standing and walking is delayed compared with children with the same age. These patients develop their typical gait pattern already influenced by obesity. In adult life, the progressive effects of obesity on joints, small feet, hypotonia and the other orthopaedic problems produce further gait deviations.

Rehabilitation programs aimed at improving hypotonia as well as at stimulating the development of motor skills, should be planned in early childhood of PWS patients. The stimulation of motor activity, through its positive action on muscle mass, physical strength and energy balance, may contribute to improve the life expectation of patients with PWS and their quality of life [29]. Appropriate rehabilitation, osteopathic treatments (to be started in early childhood), hypocaloric diet, GH therapy [30] and treatment of behavioral abnormalities, are the cornerstones of a multidisciplinary PWS patients treatment.
